# Bone Disease in HIV: Need for Early Diagnosis and Prevention

**DOI:** 10.3390/life14040522

**Published:** 2024-04-17

**Authors:** Georgios Schinas, Ioannis Schinas, Georgios Ntampanlis, Eleni Polyzou, Charalambos Gogos, Karolina Akinosoglou

**Affiliations:** 1 School of Medicine, University of Patras, 26504 Rio, Greece; georg.schinas@gmail.com (G.S.); dabanlisjr@gmail.com (G.N.); polyzou.el@gmail.com (E.P.); cgogos@upatras.gr (C.G.); 2 School of Medicine, National and Kapodistrian University of Athens, 15772 Athens, Greece; giannis.sch@gmail.com; 3 Department of Internal Medicine and Infectious Diseases, University General Hospital of Patras, 26504 Rio, Greece

**Keywords:** HIV, antiretroviral therapy, highly active, osteoporosis, osteopenia, fractures, bone, disease management, bone density, risk factors, diagnostic techniques, endocrine, patient-centered care

## Abstract

The transformation of HIV into a manageable chronic condition has unveiled new clinical challenges associated with aging-related pathologies, including bone disease. This review explores the intricate relationship between HIV, antiretroviral therapy (ART), and bone disease, highlighting the necessity of early diagnosis and preventative strategies to mitigate the increased risk of osteopenia, osteoporosis, and fractures in people living with HIV (PLWHIV). It synthesizes the current literature to elucidate the multifactorial etiology of bone pathology in this population, that includes direct viral effects, chronic immune activation, ART-associated risks, and the impact of traditional risk factors for bone loss. Through a critical examination of modern diagnostic methods, lifestyle modifications, evidence-based preventive actions, and pharmacological treatments, the necessity for comprehensive management is highlighted, along with recommendations for integrated healthcare approaches vital for achieving optimal patient outcomes. By advocating for a proactive, patient-centered, and multidisciplinary strategy, this review proposes a plan to integrate bone health into standard HIV care through active risk identification, vigilant screening, effective preventive measures, tailored treatments, and informed decision-making, in an effort to ultimately enhance the quality of life for PLWHIV.

## 1. Introduction

The transformation of HIV infection into a manageable chronic condition, as a result of effective antiretroviral therapy (ART), represents a significant modern medical achievement. However, this advancement has unveiled new clinical challenges in the form of advanced age disease in people with HIV (PWH), with bone disease being at the forefront of scientific discussion and research in the field. The compelling nature of this subject stems from the multifaceted risk factors for bone disease in HIV, which involve a combination of traditional risk factors and those uniquely associated with HIV infection and its treatment [1]. Recent studies, employing a variety of modern, state-of-the-art diagnostic techniques have confirmed altered bone microarchitecture and reduced bone strength in this population, which could be attributed to the impact of prolonged ART, the chronic inflammatory state associated with the condition, or a combination of these factors [2,3,4]. 

The ART-associated decline in bone mass has been a significant concern for many years, with a metanalysis from 2006 indicating a 2.5-fold increase in the odds of reduced bone mineral density (BMD) for individuals on ART compared to their treatment-naïve counterparts [5]. However, the prevalence of osteopenia, and osteoporosis in PWH in general, was alarmingly high in this study, with 67% of PWH exhibiting reduced BMD, and 15% meeting the criteria for osteoporosis. A more recent metanalysis on the matter, reports a more modest 2.4-fold increase in odds of osteopenia/osteoporosis in PWH and a 2.8-fold increase in odds in individuals on ART, highlighting the abundance and variety of significant pertinent risk factors in the HIV demographic [6]. Finally, a relatively large and recent comparative analysis of 1143 individuals from China found that ART-naïve patients had a lower prevalence of osteopenia (18.3%) and osteoporosis (1%) than those receiving ART (29.8% and 2.4%, respectively), illustrating the contributory role of ART in bone disease clearly [7].

Regardless of the underlying factors, bone disease-associated conditions, i.e., osteopenia and osteoporosis, are significantly relevant from a clinical perspective, as they directly increase the risk of fractures, complicating the clinical management of individuals with HIV [8]. A meta-analysis comprising 21 studies has solidified the magnitude of this issue, revealing a pooled prevalence of fracture of 6.6% in PWH, a percentage that is nearly double that of the general population [9]. This finding is further corroborated by data from the prospective, US-based HIV Outpatient Study (HOPS), assembled during a period of 17 years, which demonstrated a fracture prevalence of 7.5% among 6763 participants. Of note, 26.8% of these fractures were defined as osteoporotic, based on bone location [10]. The implications of these findings are profound, as fractures in the context of HIV are associated with increased all-cause mortality as demonstrated by the same study’s comprehensive results [hazard ratio (HR) 1.48, 95% CIs: 1.15–1.91], underscoring the critical need for preventative strategies aimed at preserving bone health.

With the aging of the HIV population, comprehensive management strategies become increasingly important to enhance the overall quality of life, especially considering the interconnectedness of various aspects of care in treated and multi-treated individuals living with HIV. Through a critical examination of current research perspectives and clinical practices, this review aims to establish a foundation for future investigations and offer guidance on the integrated healthcare approaches required to address bone health within the context of HIV management by prioritizing early diagnosis and prevention.

## 2. Understanding Bone Disease in PLWHIV and Establishing the Framework for Interventions 

### 2.1. HIV and Bone Loss Pathophysiology

The pathophysiology of bone disease in HIV involves both direct viral effects and immune system interactions that lead to bone demineralization, increased bone turnover, and eventually bone loss. To begin with, HIV can infect osteoblast precursors, disrupting normal bone formation processes and leading to impaired osteogenesis. The p55-gag and REV viral proteins, in particular, have been shown to reduce the expression and activity of RUNX2, a critical transcription factor for osteogenesis, and to increase the activity of PPARgamma, a transcription factor associated with adipogenesis, suggesting a shift in the differentiation of mesenchymal stem cells (MSCs) away from osteoblasts and toward adipocytes [11]. More recently, other viral proteins, namely Nef and Tat, were demonstrated to modulate MSCs’ development and alter osteoblast development, in ART-naïve individuals, thereby putting the direct viral effects on bone formation into the spotlight [12]. Moreover, p55-gag and gp120 have been found to reduce calcium deposition and alkaline phosphatase (ALP) activity and decrease the levels of bone morphogenetic protein (BMP)-2 and -7, as well as the receptor activator of nuclear factor kappa-Β ligand (RANK-L) [13]. The latter observation is of significant importance because it exacerbates the situation. Specifically, the direct viral interference with bone formation is augmented by the virus’s enhancement of RANKL-mediated signaling pathways, alongside a simultaneous reduction in osteoprotegerin (OPG) activity. This dual action shifts the equilibrium towards increased bone resorption, a process simulated first in an HIV animal model [14]. Subsequently, increased B-cell RANKL/OPG expression ratio was directly linked to decreased BMD of the hip and femoral neck in a human study as well [15]. These findings are consistent with the observation that HIV proteins, such as Tat and gp120, upregulate bone resorption, by inducing the release of cytokines, thereby influencing immune-mediated mechanisms driving osteoclastic function [16].

The complex interaction between the virus and the host’s immune system, particularly chronic immune activation and inflammation, can lead to increased osteoclast activity and bone resorption. This immune dysregulation exerts its actions on bone through established pathways of inflammation involving cytokine activation and increased circulation, as in the case of TNF-α and IL-6, which have been found to be elevated in HIV-infected individuals and are known to influence bone metabolism [17]. A 48-week prospective cohort study that compared changes in hip and spine BMD between ART-naive PWH and healthy controls matched by age, sex, and race revealed that higher IL-6 measurements were independently associated with progression to osteopenia or osteoporosis in the HIV-infected group [18]. Furthermore, chronic T-cell activation has been shown to lead to an elevated RANKL/OPG ratio, thus contributing directly to bone loss. A case-control study, conducted by the group that had previously reported the same imbalance in B-cell populations, demonstrated that the T-cell RANKL/OPG ratio significantly correlates with reduced BMD scores at the hip, lumbar spine, and femur neck in ART-naïve PWH with CD4+ T-cell counts ≥ 200 cells/μL. Conversely, this correlation was absent in individuals with severely decreased CD4+ counts (<200 cells/μL), indicating a complex impact of T-cell dynamics on bone health, dependent on the level of CD4+ depletion [19]. In summary, HIV immune-mediated systemic responses seem to stimulate osteoclastogenesis and disrupt the bone remodeling process, leading to an increased risk of osteopenia, osteoporosis, and ultimately, fractures [20].

Compounding this systemic impact, T-cell depletion within the gut-associated lymphoid tissue (GALT) shortly after HIV infection leads to increased barrier permeability and microbial translocation, elevating lipopolysaccharide (LPS) levels [21]. This sequence of events further catalyzes a shift towards a pro-inflammatory cytokine milieu [22], potentially enhancing osteoclastogenesis and bone resorption [23]. Emerging evidence suggests that alterations in the gut microbiome itself may play a crucial role in this process. A recent cross-sectional analysis has shown that women with chronic HIV infection exhibit unique gut microbiota compositions and plasma metabolite profiles directly associated with low BMD [24]. These profiles, featuring changes in bacterial genera and metabolites linked to bone health, suggest that the state of the gut microbiome could significantly influence bone degradation observed in PWH.

### 2.2. Impact of ART on Bone Health

The role of ART in bone health cannot be overlooked. Its overall impact seems to be a multifaceted issue that encompasses inherent bone loss risk, the patient’s general health condition and comorbidities, as well as specific effects by ART classes, initial bone loss upon ART initiation, and long-term implications [23].

In general, however, ART has been associated with reduced BMD, increased bone turnover and elevated osteoporosis risk in PWH [25,26]. Specific antiretroviral agents, such as tenofovir disoproxil fumarate (TDF)-containing regimens, have been linked to decreased BMD in several randomized clinical trials (RCTs) and observational studies [27,28,29]. The latest metanalysis suggests that TDF causes greater decreases in BMD than comparators when used for pre-exposure prophylaxis (PrEP), HIV treatment, and HBV treatment, with no increased fracture risk [30]. This effect was more pronounced when used in HIV treatment regimens, rather than in PrEP, a finding consistent across all BMD measurement sites. The mechanism through which TDF affects bone turnover may include renal tubular dysfunction (Acquired Fanconi’s Syndrome) [31], and phosphaturia in particular, which has been associated with lower BMD in PWH on TDF-based therapy [32]. Notably, Fanconi’s syndrome reversal has been reported upon discontinuation of TDF [33], providing a credible basis for the observed beneficial ‘switch-effect’ from TDF to tenofovir alafenamide (TAF)-based ART. This switch has been demonstrated to improve BMD in RCTs [34,35], and is now recognized as an established practice in clinical settings, although a benefit in terms of reduced fracture risk has not yet been proved. Other classes of antiretrovirals, such as protease inhibitors (PIs) and nucleoside analog reverse-transcriptase inhibitors (NRTIs) have also been associated with reduced BMD in RCTs, as ART-regimen components [27,28] and independently [36], as well as increased osteoclast activation and proliferation in in vitro studies [37,38]. PIs, in particular, are implicated in reducing the RANKL/OPG ratio and inhibiting osteoblastic activity, thus promoting bone resorption [39]. On the other hand, studies have ascertained the bone-friendly profile of integrase strand transfer inhibitors (INSTIs), especially Dolutegravir, which is associated with either BMD improvement in virologically suppressed patients or minimal impact on BMD, presenting a favorable comparison to PIs and NRTIs [40].

#### 2.2.1. Short-Term Effects of ART on Bone

Upon the initiation of ART, a notable decrease in BMD is frequently observed, with reductions typically ranging from 2% to 6% within the first two years of treatment. This period of initial bone loss is often followed by a phase of stabilization or potential increase in BMD [41]. A study by the AIDS Clinical Trials Group, involving 4640 participants across 26 randomized ART studies with a median follow-up of 5 years, found an initial increase in fracture rates to 0.53 per 100 person-years in the first 2 years after ART initiation, which subsequently decreased to 0.30 per 100 person-years. This suggests a short-term elevated risk of fractures following ART initiation that stabilizes over time, with factors like smoking and glucocorticoid use, rather than specific antiretrovirals, associated with increased fracture risk [42]. Therefore, this short-term loss of bone density represents a critical period for early evaluation and intervention, potentially by addressing modifiable factors, to prevent further bone loss and mitigate future fracture risk. 

The exact mechanisms underlying these early changes are not entirely understood but may involve processes such as T-cell repopulation and immune reconstitution inflammatory syndrome (IRIS). These factors have been linked to increased bone turnover and subsequent BMD loss in immunocompromised mice [43]. Importantly, the extent of immune reconstitution in terms of CD4+ count, has been associated with improvements in bone resorption markers in humans, as observed in a study over a 24-week period following ART initiation [44]. However, it is interesting to note that while low baseline CD4+ counts have been independently associated with greater BMD loss, significant increases in CD4+ count over 16 weeks post-initiation have not been directly correlated with BMD improvements [45]. This observation points to the complexity of ART’s perceived impact on bone through immune enhancement. In this context, the findings from the START Bone Mineral Density Substudy provide valuable insights. The study demonstrated that immediate ART initiation (with CD4 > 500 cells/μL) resulted in greater BMD declines at both the hip and spine over 2.2 years of follow-up, compared to deferred ART initiation (to CD4 < 350 cells/μL) [41]. These results bring attention to the dual nature of ART’s effects, suggesting the need for a nuanced approach in managing ART, especially considering the long-term implications for bone health.

#### 2.2.2. Long-Term Effects of ART

Over the long term, the impact of ART on bone health appears to stabilize, with BMD measurements of the spine and hip converging following 3 years of treatment between treated and treatment-naïve individuals [41]. Significant ART-related bone loss, in particular, seems to subside after the first full year of steady ART in previously treatment-naive PWH [46]. Nonetheless, bone health appears to be a persistent concern, with ongoing risks of osteopenia and osteoporosis among multi-treated individuals. A case-control study investigating long-term ART’s effect on bone microstructure in patients matched for age and BMI, with a median of 18.2 years of reportedly successful ART, revealed significant microstructural alterations in both trabecular and cortical bone compartments among HIV-positive men between the ages of 60 and 70 years old [47]. Notably, these findings persisted despite adequate vitamin D supplementation and were associated with higher markers of bone resorption. Multivariate analyses further identified lower physical activity, and reduced estradiol levels as significant determinants of impaired bone architecture. Another investigation carried out to investigate specific risk factors related to bone alterations in individuals with long-term HIV treatment, specifically between the ages of 50–70 years old, revealed malnutrition and physical inactivity as significant contributors. The study also noted that the use of TDF in conjunction with PIs exacerbates the risk of trabecular bone deterioration [48]. 

As to more specific aspects of long-term therapy, the evidence on the impact of CD4+ count on bone and fracture risk remain heterogenous. Most studies report no association between CD4+ counts and bone quality indicators over the long-term [48,49]. Nonetheless, the HOPS Study’s first results from the 2000–2008 cohort, pointed to an association between nadir CD4 cell counts of less than 200 cells/microL and a higher risk of fractures (aHR 1.60, 95% CIs: 1.11–2.31, *p* < 0.01) [50]. Yet, continuous ART has been linked to a decline in BMD and a possible increase in fractures compared to intermittent ART, guided by CD4 cell count, suggesting an overall adverse effect of prolonged ART on bone health [51]. In summary, although the short-term effects of ART initiation on bone health are better understood and can be more readily targeted for interventions, prolonged ART use may still present a continuous risk for osteopenia, osteoporosis, and fractures. This realization hints at the importance of a comprehensive management strategy, which includes careful monitoring and interventions designed to maintain bone health in individuals undergoing long-term treatment.

### 2.3. Modifiable Risk Factors Contributing to Bone Disease in HIV

The interplay between HIV infection and bone health is significantly influenced by a spectrum of modifiable risk factors, including vitamin D deficiency, calcium insufficiency, lifestyle factors such as nutrition, smoking and alcohol use, and reduced physical activity. Understanding these factors is crucial for developing targeted interventions to mitigate bone disease in this population.

#### 2.3.1. Vitamin D Deficiency and Malnutrition

Vitamin D plays a pivotal role in calcium homeostasis and bone metabolism, with deficiency in this nutrient being a well-recognized risk factor for bone loss and osteoporosis. High prevalence rates of vitamin D deficiency have been reported among PWH, regardless of treatment status [52]. A comparative study from the United States reported that 70.3% of PWH not on vitamin D supplements had serum 25-hydroxyvitamin D levels below 30 ng/L (<75 nmol/L), a figure slightly less than the 79.1% prevalence observed in the general U.S. population [53]. Notably, risk factors for vitamin D insufficiency or deficiency included black race, Hispanic ethnicity, and lack of exercise, with black race being associated with a significantly higher adjusted odds ratio (aOR, 4.51; 95% CI, 2.59–7.85). This observation is further supported by another US study which found that 69% of patients with HIV on ART, 61% of whom self-identified as Black, exhibited very low 25-hydroxy-vitamin D levels (<20 ng/L [<50 nmol/L]), a rate not significantly different from a matched control group without HIV [54]. Furthermore, hypocalcemia, which is closely related to vitamin D deficiency, has been observed at higher rates in patients with HIV (6.5%) compared to those without (1.1%), with vitamin D deficiency identified as the causative factor in most cases [55]. 

It becomes evident that vitamin D deficiency may not be solely attributed to nutritional factors or HIV infection per se but could also reflect broader population trends, particularly in certain demographics such as the Black population, where low vitamin D levels are inherently more prevalent. Nonetheless, the nutritional aspect should not be underrated in the HIV demographic. Malnutrition, as determined by nutritional assessments, is associated with microstructural changes in the cortical bone compartment of long-term treated individuals living with HIV [48]. Moreover, indirect indicators of malnutrition, such as low body weight and decreased albumin levels, have been linked to diminished BMD in PWH who have been HIV-positive for a median duration of 7.7 years on ART [49]. Finally, in the HIV Outpatient Study (HOPS) cohort, a severely low BMI (<18.5 kg/m^2^) emerged as the most significant independent predictor of fragility fractures, with an adjusted hazard ratio (aHR) of 3.72 (95% confidence interval [CI], 1.14–12.09) [50].

#### 2.3.2. Smoking and Alcohol Use

Lifestyle factors such as smoking and alcohol use are well-documented contributors to bone loss and fracture risk in the general population [56,57] and can have similar impacts on individuals with HIV. Addressing these modifiable risk factors through lifestyle interventions could play a significant role in reducing the risk of osteopenia and osteoporosis in this population. 

Associations between smoking and reduced BMD in PWH have been well established [58,59]. Additionally, smoking has been described as an independent risk factor (aHR 1.3, 95% CI: 1.1–1.5) for fracture incidence in the HIV demographic by a recent metanalysis [9]; the prevalence of smoking among PWH being 2–3 times higher than that of the general population [60,61]. In the United States, the rate of smoking within the HIV-positive community is estimated to be between 34–47%, highlighting an urgent need for targeted smoking cessation interventions [62]. Smoking cessation has been demonstrated to increase BMD in vulnerable smoker groups like post-menopausal women [63] and has been linked to increased bone formation markers in both human and non-human subjects [64]. In the HIV population, smoking cessation offers the added benefit of potentially improving body composition, which can be particularly beneficial for individuals experiencing lipodystrophy as a result of ART. In fact, quitting smoking can lead to a more significant increase in BMI in PWH compared to the general population. A specialized smoking cessation program for PWH observed a mean BMI increase of 2.3 kg/m^2^ three months post-cessation among participants, a change substantially more pronounced than that seen in the general population following smoking cessation [65]. This finding is particularly relevant given that low BMI and body weight, in general, has been identified as a significant predictor of low BMD IN PWH, as discussed before. 

The relationship between alcohol abuse and ART adherence may also complicate the clinical management of HIV in general, leading to worse treatment outcomes [66]. In the context of bone-specific outcomes, while alcohol abuse has been associated with fragility fractures in an HIV veteran cohort (HR 1.80, 95% CI: 1.50–2.17) [67], a comprehensive 3.5-year prospective cohort study of PWH with substance use disorder found no significant association between alcohol consumption and changes in BMD at the femoral neck, total hip, or lumbar spine, nor with >6% annual decrease in BMD at any site or incident fractures [68]. This suggests the relationship between alcohol use and bone health in PWH may be influenced by factors not solely related to changes in BMD. Diving deeper into the pathophysiology of the issue, a cross-sectional analysis revealed that PWH engaging in at-risk alcohol consumption exhibited significantly lower serum levels of osteocalcin, a marker of bone formation, indicating a suppression of bone formation activity independent of systemic oxidative stress levels, as measured by relevant markers [69]. Furthermore, a prospective cohort study found that higher alcohol intake is significantly associated with lower levels of procollagen type 1 N-terminal propeptide (P1NP), another bone formation biomarker, further confirming the negative impact of alcohol on bone formation in PWH with substance use disorder [70]. Corroborating these findings, another study within the New Orleans Alcohol Use in HIV cohort demonstrated that alcohol use inversely correlates with both osteocalcin and P1NP levels, with these effects being more pronounced in individuals over 50 years of age and in postmenopausal women, highlighting the compounded risk in these subpopulations [71]. Additionally, the study noted positive correlations between serum sclerostin levels and alcohol use, suggesting a potential pathway through which alcohol may exert its osteopenic effects. Given the high prevalence of alcohol use disorders among PWH, estimated at 29.80% and highly prevalent in developed countries [72], the need for systematic screening and management of potential abuse, from the perspective of preserving bone health, is imperative.

### 2.4. Comorbidities Impacting Bone Health

Several comorbidities prevalent among PWH can further complicate bone health. Among these, metabolic syndrome components such as insulin resistance and type 2 diabetes (T2D) which are highly prevalent among PWH [73,74], non-alcoholic fatty liver disease (NAFLD), as well as hypogonadism and hepatitis C virus (HCV) co-infection. Moreover, 68% of PWH on ART have been reported to receive at least one prescription medication. The potential impact of certain non-ART medications, such as opiates, glucocorticoids, and certain anticonvulsants, has to be acknowledged as they are known contributors to bone density loss. Substance abuse, catabolic steroids use, and SSRIs have been associated with BMD loss and fragility fractures, respectively in PWH [49,50].

#### 2.4.1. Metabolic Syndrome Elements

Insulin resistance and T2D have been identified as risk factors for osteoporosis and fractures in the general population [75,76,77]. In the HIV-positive demographic, impaired glucose tolerance affects approximately 25–35% of patients, while 2–7% may develop diabetes [78]. Diabetes has been reported as an independent risk factor for the incidence of fractures in PWH (aHR, 1.62; 95% CI, 1.00–2.64) [50]. However, the interplay between glucose metabolism and bone health may not be as straightforward as it seems. In a cross-sectional study of about 100 PWH, aged 50 years or older on ART, a high frequency of insulin resistance was identified (68.2%) among participants, while the prevalence of osteoporosis was 19%. Interestingly, participants with osteoporosis had lower insulin resistance measures, suggesting an inverse relationship between insulin resistance and osteoporosis severity. However, this association appeared to be influenced by BMI, which was significantly lower in the osteoporotic subgroup, as were triglyceride levels [79]. 

On the other hand, NAFLD, a condition often observed in long-term TDF-based antiretroviral therapy recipients, has been directly linked to decreased BMD in HIV. A case-control study involving 89 PWH on long-term TDF-based therapy revealed that the prevalence of osteopenia and osteoporosis was notably higher in the NAFLD subgroup, with incidence rates of osteopenia at 42.86% vs. 25.93%, and osteoporosis at 17.14% vs 3.70% for those with and without NAFLD, respectively [80]. The odds of experiencing decreased BMD with NAFLD, were calculated at 4.49 (95% CI: 1.42–14.15).

#### 2.4.2. Hypogonadism

Hypogonadism, an endocrine disorder commonly associated with HIV, affects about 20% of HIV-positive males [81]. This condition contributes to the risk of bone loss, in a similar way to menopause causing hormonal imbalance primarily characterized by decreased circulating estradiol levels and increased sex-hormone binding globulin (SHBG) levels. A cross-sectional study involving 168 men on stable cART, with a median age of 53 years, explored this relationship in depth. Hypogonadism was present in 26.2% of cases. The prevalence of osteoporosis and osteopenia in this cohort was notably high at 87.5%, with vertebral fractures detected in 25% of patients, while elevated Follicle Stimulating Hormone (FSH) levels adversely affected femoral BMD. Higher SHBG levels were predictive of vertebral fractures and correlated inversely to BMD in the spine and femur [82]. Another cross-sectional study on the matter, compared 80 male patients, who did not exhibit wasting syndrome and were not on highly active antiretroviral therapy (HAART), with 20 healthy male controls. In this cohort, patients with hypogonadism—which accounted for 40% of the participants—were found to have significant alterations in markers of bone health. Specifically, hypogonadal patients exhibited significant decreases in serum osteocalcin, a marker of bone formation, and increased urinary excretion of crosslinks, indicators of bone resorption. Furthermore, this subgroup also demonstrated a statistically significant reduction in BMD at all measured sites [83].

#### 2.4.3. Hepatitis C Virus (HCV) Infection

Infection with HCV has been associated with reduced BMD and an increased fracture risk, both in the presence and absence of HIV infection [42,84,85]. A comprehensive study of the Medicaid population of multiple US states, conducted during a period of 7 years, highlighted that the incidence of hip fracture was notably higher in patients coinfected with HIV and HCV (3.06 events per 1000 person-years) compared to those with HCV alone (2.69 events per 1000 person-years), HIV alone (1.95 events per 1000 person-years), and uninfected individuals (1.29 events per 1000 person-years), suggesting that the coexistence of these conditions may synergistically impair bone health [86]. Importantly, however, the eradication of HCV does not appear to increase BMD in co-infected individuals, nor does it correlate with plasma concentration changes of soluble RANKL or OPG [87].

## 3. Preventative and Therapeutic Strategic Planning

**Figure 1:**
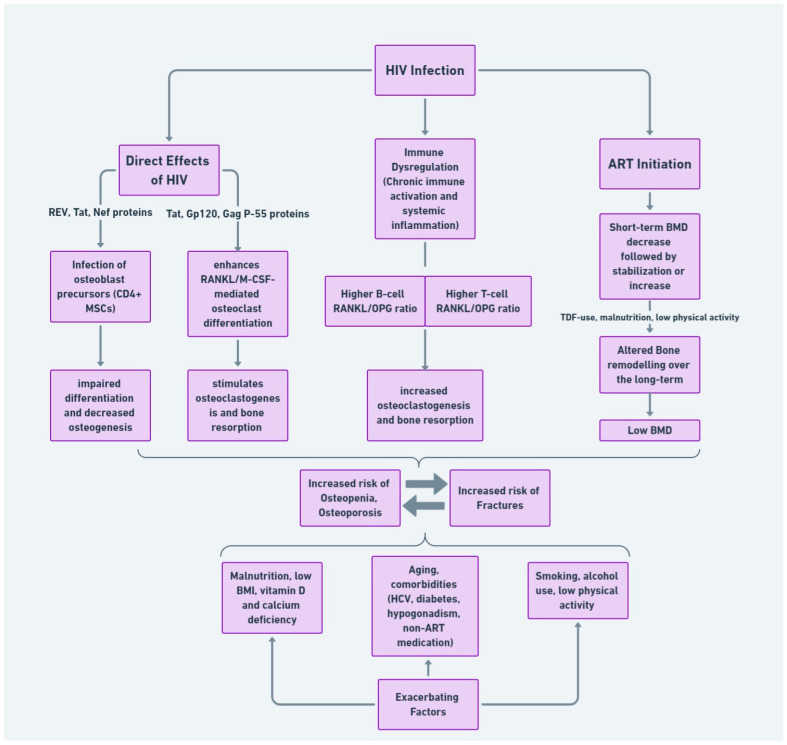
Pathophysiology of bone disease in HIV infection.

Given the multifactorial nature of bone disease in PWH, a holistic approach to management is required. The intricate relationship between immune response, ART, and modifiable factors affecting bone health necessitates multiple clinical considerations, with attention given to both the direct and indirect factors contributing to bone disease. Figure 1 succinctly encapsulates the complex interplay of these elements, setting the stage for the formulation of a unified management plan. With this comprehensive understanding as the backbone, we will consequently examine the diagnostic assessments and interventions required to address bone disease in HIV.

### 3.1. Diagnostic Assessments for Early Detection of Bone Disease

The early detection of bone disease in individuals living with HIV is critical for timely intervention and management. Diagnostic assessments, primarily BMD screening and evaluation for secondary causes, are foundational elements of a concentrated approach to bone health in this population.

#### 3.1.1. Bone Mineral Density Screening

Screening for osteoporosis is widely advocated for all postmenopausal women and men over 50 years of age with HIV, as HIV infection is considered an additional risk factor for osteoporosis [88] Factors prompting earlier screening may include a low BMI or lean body mass, smoking, alcohol abuse, hypogonadism, chronic glucocorticoid use, kidney disease or early menopause [1].

The gold standard for BMD assessment, dual-energy X-ray absorptiometry (DEXA), provides a reliable, quick, and low-radiation method for diagnosing osteoporosis despite some limitations, such as sensitivity to bone size and potential for artifact interference [89]. However, its availability in certain settings, such as low-middle income settings, is limited, prompting the use of alternative diagnostic tools to guide the decision-making process for screening and treatment, such as the Fracture Risk Assessment Tool (FRAX) [90]. 

FRAX provides a 10-year fracture risk estimation using clinical risk factors, with or without BMD data, including age, gender, and lifestyle factors (https://frax.shef.ac.uk/frax/, accessed on 1 April 2024). However, FRAX does not incorporate HIV status as a covariate, nor has it been validated in the HIV population. As a matter of fact, utilizing the FRAX model for the assessment of fracture risk for PWH has shown mixed effectiveness. A recent study investigating FRAX score’s role in 774 PWH over 50 years old, suggests a significant discrepancy between FRAX scores and DXA scan results. Specifically, the prevalence of osteoporosis in the cohort was 12.2%, and osteopenia was 63.7%, yet FRAX major was >10% in only two patients, and 98.9% of patients with osteoporosis had a normal FRAX score [91]. Consequently, the European guidelines make special recommendations on the use of the FRAX tool to refine fracture risk prediction in the HIV population. To begin with, they acknowledge the potential underestimation of risk in PWH and recommend considering HIV as a cause of “secondary osteoporosis” in the calculation. Additionally, the guidelines recommend adding the Trabecular Bone Score (TBS), derived from DXA scan results, to enhance the accuracy of FRAX risk predictions [92]. A study from 2020 has, in fact, investigated the aforementioned recommendations in a clinical context. In a longitudinal assessment of 217 PWH, with a mean age of 45.8 years, who had previously undergone DXA screening, the FRAX was calculated both with and without the inclusion of femoral neck BMD data, and the “secondary osteoporosis” indication checked for all participants [93]. The results revealed that 61% of individuals exhibited low BMD, yet 98.5% of these were not identified as candidates for DXA screening based on current FRAX thresholds. Notably, among individuals aged under 50, 23% had low BMD, but none were deemed candidates for DXA screening. Incorporation of BMD data into the FRAX calculation led to an increase in FRAX results by 50–100%, with only 1%, however, exceeding the threshold for recommended DXA screening. Therefore, the need for adjusted screening criteria or the development of HIV-specific risk assessment tools becomes evident. 

The optimal interval for BMD rescreening in PWH is a matter of debate. In the general population, determining the interval for subsequent BMD screenings based on initial BMD findings is recommended. Specifically, a rescreening period of about 15 years for women who have normal bone density or mild osteopenia, 5 years for those with moderate osteopenia, and annually for women exhibiting advanced osteopenia is suggested [94]. A study within the HIV population proposed slightly different intervals based on T score tertiles, specifically 1 to 2 year interval for individuals in the lowest T score tertiles and interval of 6 years or more for those in the highest tertiles, as the time to progression to osteoporosis was calculated at over 8 years for those with mild to moderate osteopenia and 3.2 years for those with advanced osteopenia [95].

#### 3.1.2. Future Directions on Early Diagnosis

In an innovative study from Italy, researchers developed an osteopathy prediction model, aiming to improve fracture risk assessment in PWH [96]. Recognizing the limitations of existing models like FRAX, which do not account for HIV-related factors, this model incorporates both traditional and HIV-specific determinants of bone health. Specifically, the model integrates the intrinsic effects of HIV infection per se, assigning hazard ratios (HR) of 1.10 for men and 1.00 for women, to pinpoint the differential impact of HIV on fracture risk by gender. Additionally, it addresses the impact of particular ART components such as TDF and PIs, thereby promising a more accurate depiction of fracture risk in the HIV-treated population. For instance, the model specifies an elevated HR for fracture risk due to TDF use at 1.11, highlighting a modest yet significant risk increase. PIs are identified with a similar HR of 1.10, suggesting a comparable risk enhancement. Notably, the concurrent application of TDF and PIs leads to further risk augmentation, with an HR of 1.16, marking the most pronounced risk escalation among the drug combinations included. The development of the prediction model was based on a thorough literature review and validated through cohort-level analysis, aiming to ensure its applicability and accuracy. By addressing the limitations of FRAX, this effort on an HIV-specific clinical aid tool paves the way for improved clinical outcomes through personalized risk assessment.

Building on this discussion, an exploration of alternative imaging modalities is warranted. Quantitative computed tomography (QCT) can provide detailed analysis of both bone compartments -cortical and trabecular-, providing insights into the microstructure and is useful in research contexts for predicting fracture risk [97]. It has been used in multiple studies involving PWH, demonstrating utility particularly in young and elderly patients, who may display cortical alterations [25]. Magnetic resonance imaging (MRI) offers a non-radiative option for characterizing bone composition, making it an appealing option for younger individuals and is recommended in cases of suspected osteoporotic fractures, as it can reflect physiologic alterations based on signal intensity [98]. Finally, quantitative ultrasound (QUS) is a practical and accessible method, especially in resource-constrained environments, which correlates well with BMD and can aid in identifying patients at risk of fractures [99,100]. Utilizing QUS to assess BMD in PWH has been investigated by some studies with mostly positive results [101,102]. To summarize, these three alternative assessments could potentially be valuable for specific settings or subpopulations, complementing the diagnostic aspect of bone health in PWH.

#### 3.1.3. Evaluating for Secondary Causes

If osteoporosis is diagnosed, it is essential to investigate and address secondary causes. A comprehensive laboratory evaluation should include markers such as 25-hydroxy-vitamin D, calcium and phosphorus levels, renal function tests, liver enzymes, bone turnover markers, parathyroid hormone (PTH), thyroid stimulating hormone (TSH), and sex hormones. Additional or more specialized testing for conditions like multiple myeloma, celiac disease, and Cushing syndrome may be warranted based on clinical suspicion. 

The intersection of chronic kidney disease (CKD) and osteoporosis in PWH requires special attention. The prevalence of CKD in the HIV population has been reported to be as high as 15%, with up to 40% experiencing subclinical renal tubular alterations that can disrupt the renal-bone axis, leading to significant BMD loss [103,104]. Additionally, the prevalence of osteoporosis is reportedly high among HIV-positive renal transplant recipients, with a study indicating a prevalence rate of 20% in this group [105]. Of particular importance in this context is the use of TDF within ART regimens, which poses a dual threat by contributing to nephrotoxicity and, indirectly, osteoporosis, by disrupting calcium and phosphate balance [106]. A targeted approach in managing osteoporosis’ risk within this demographic is imperative, advocating for the integration of specialized renal disease evaluation, including kidney biopsy, into osteoporosis care protocols, alongside the established laboratory evaluations for osteoporosis secondary causes.

### 3.2. Prevention and Management of Bone Disease

Managing low BMD in HIV populations poses unique challenges due to the younger age of these patients compared to the traditional osteoporosis cohort [107]. General recommendations, including lifestyle interventions and pharmacological treatment, are generally consistent with those for the non-HIV population. The goal is to maintain 25-hydroxy-vitamin D levels conducive to bone health while minimizing the risk of fracture through comprehensive management and screening, with pharmacologic treatments such as bisphosphonates reserved for those with a substantial risk of bone disease. Treatment is recommended for postmenopausal women and men over 50 with a T-score ≤ −2.5, patients with a history of fragility fractures, or those with low bone mass and a significant 10-year fracture probability as per FRAX thresholds.

#### 3.2.1. Vitamin D Supplementation

For individuals with HIV, who are at an increased risk for bone disease, the management of vitamin D levels is a critical aspect of care. Replacement and/or supplementation of vitamin D is recommended for those with insufficiency and conditions such as osteoporosis, osteolamalacia or increased PTH, with a re-testing suggestion after 6 months of intake. Targeting a 25-hydroxy-vitamin D level of at least 30 ng/mL is generally recommended for PWH with osteoporosis. This target is higher than the recommended threshold for bone health adequacy (above 20 ng/mL), considering the elevated fracture risk in the HIV population. Achieving the recommended 25-hydroxy-vitamin D levels above 30 ng/mL often necessitates supplementation, as dietary intake alone is usually insufficient. 

However, while clinical trials provide some guidance, especially in the context of TDF-based regimen therapy, individualized assessment and monitoring are essential to ensure optimal outcomes. For instance, one clinical trial indicated that individuals beginning efavirenz-TDF-emtricitabine therapy experienced less bone loss at the hip and spine when supplemented with high-dose vitamin D (4000 IU daily) and calcium carbonate (1000 mg daily) over 48 weeks [108]. The authors suggest that such supplementation may be an effective intervention to prevent bone loss in patients with HIV initiating ART. Another study demonstrated that young males on TDF regimens benefited from a monthly high-dose vitamin D supplement (50,000 IU), which resulted in improved lumbar spine BMD when combined with a daily multivitamin containing vitamin D and calcium [109]. Other approaches may include nutritional support and patient education on improving dietary habits. A study proposed that medical nutrition therapy can improve the diet to promote bone health in people living with HIV, suggesting a significant increase in the intake of nutrients like calcium and vitamin D, and an increase in exercise length after the intervention [110]. Among calcium-rich foods, yogurt intake has been reported as a protective predictor of lumbar spine BMD in PWH [111].

#### 3.2.2. Pharmacological Treatment

Bisphosphonates stand as the cornerstone of pharmacologic osteoporosis treatment. As with all treatments in the HIV population, individualized care and balancing of benefits versus risks are key to achieving optimal bone disease outcomes. Careful consideration is, therefore, necessary for the use of these medications in the HIV population, given their potential for adverse effects, such as the feared osteonecrosis. Nevertheless, the efficacy of bisphosphonates in treating osteoporosis in PWH has been explored through several clinical trials and two meta-analyses that suggest overall positive results and a good safety profile [112,113].

To specify further, in a 48-week prospective RCT of 31 individuals showed that alendronate (70 mg weekly), in conjunction with vitamin D (400 IU daily) and calcium (1000 mg daily as calcium carbonate), led to a significant 5.2% increase in lumbar spine BMD in HIV-infected subjects on ART [114]. This increase was greater than the 1.3% improvement observed in subjects who received only vitamin D and calcium supplementation. Importantly, no serious adverse effects were reported. In another randomized, placebo-controlled multicenter trial involving 82 subjects (71% men, with a median age of 48 years) who were receiving stable ART, the combined use of alendronate with calcium and vitamin D supplementation was shown to effectively improve BMD, demonstrating significant improvements at the lumbar spine, total hip, and trochanter [115]. Although, there were trends towards increased BMD in the group receiving only calcium and vitamin D supplementation, the addition of alendronate resulted in a more pronounced enhancement of bone density. Again, alendronate was well-tolerated, with no significant adverse events associated with its use.

Administered annually, zoledronic acid has also been shown to increase BMD in PWH with osteopenia or osteoporosis in multiple RCTs with various follow-up periods spanning from 24–36 months [116,117]. Of particular interest is an RCT of 43 individuals on HAART with osteopenia, which revealed significant BMD increases in the lumbar spine (8.9 percent) and total hip (3.8 percent) with zoledronic acid over a period of 2 years [118], with benefits persisting for up to 11 years post-treatment in a treatment subgroup [119]. Moreover, a multi-center, open-label study proved that two consecutive doses of zoledronic acid were superior to TDF-switching at improving BMD among individuals on TDF for a mean duration of 5.9 years [120]. Safety profile was comparable between the two groups. 

As for prophylactic purposes, a phase 2, placebo-controlled trial explored the efficacy of a single dose of zoledronic acid in preventing ART-induced bone loss in 63 ART-naive adults with HIV [121]. The study found significant reductions in bone resorption markers and modest improvements in BMD in the intervention group compared to placebo over the first 48 weeks of ART. The decrease in bone resorption markers was evident from 12 weeks (73% reduction; *p* < 0.001) and persisted until week 48 (57% reduction; *p* < 0.001). Complementing these findings, more focused research into the drug’s mechanism of action revealed its capacity to effectively reduce RANKL expression [122] and mitigate osteoporosis in PWH on tenofovir [123].

Alternative treatments like denosumab may be beneficial for certain patients, especially when bisphosphonates are contraindicated or ineffective. A 12-month, open-label, prospective study showed that both denosumab and zoledronate improved BMD in 23 males with HIV, with no significant difference in lumbar spine BMD change or adverse events between the two groups [124].

#### 3.2.3. Physical Activity and Exercise

The relationship between physical activity and BMD in PWH is becoming increasingly recognized as a critical area for intervention to combat bone loss [125]. A secondary analysis of data from the SATURN-HIV study, consisting of 147 individuals (78% male, median BMI 26.72 kg/m^2^) on stable ART, highlighted that physical activity of increased intensity was associated with higher BMD at the total hip and lumbar spine, suggesting that moderate-to-high intensity exercise could attenuate bone loss in PWH [126]. Moreover, in a prospective study of 120 post-menopausal women on ART, physical activity was positively associated with left femoral neck BMD [127]. Strength training, in particular, has been shown to improve BMD in PWH on stable ART as well as those exhibiting lipodystrophy [128,129].

Examining the issue from another perspective, an RCT on eugonadal men with AIDS wasting revealed that testosterone administration, rather than resistance training, over a three-month period led to an increase in lumbar spine BMD [130]. To supplement these findings, testosterone use among men living with HIV was associated with higher BMD, particularly in those with virologic suppression [131]. These findings could, thus, indicate the potential of anabolic strategies, including testosterone therapy, to address bone loss in PWH and AIDS. Within the scope of physical activity and its potential impairment, the evaluation of fall risk could also be a crucial aspect of comprehensive care, aiming to further mitigate the risk of fractures. Multiple comorbidities as well as many medications have been linked to an increased fall risk among PWH and should be taken into account [132].

### 3.3. Comprehensive Bone Health Management Plan

The approach to managing bone health in PLWHIV is grounded in a comprehensive framework that integrates the identification of risk factors, vigilant screening, and monitoring practices, along with lifestyle and pharmacological interventions. Incorporating these elements into a coherent action plan allows for a dynamic response to the inherent challenges of HIV clinical management, with the ultimate goal of enhancing patient outcomes and quality of life through informed, proactive care.

**Figure 2:**
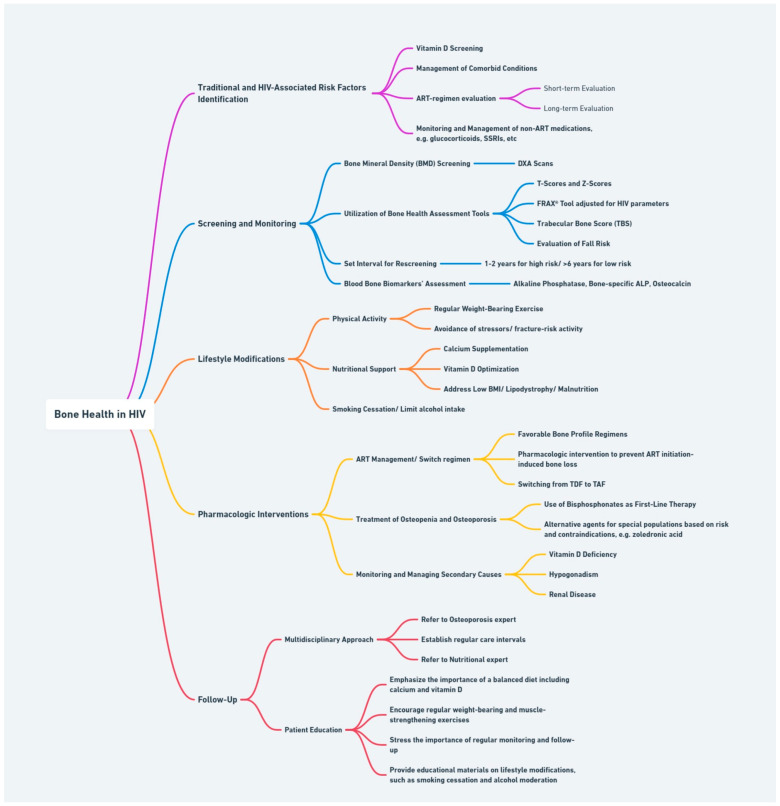
Comprehensive management strategy for bone health in HIV-infected individuals.

Figure 2 outlines this multi-tiered strategy, capturing the core of our proposed methodology, mapping the journey from identifying risk factors to implementing interventions and ensuring follow-up. This comprehensive plan of action is rooted in evidence-based practices, designed to mitigate the risk of osteoporosis and fracture among the vulnerable HIV population. At the forefront of our strategic approach are proven interventions targeted at improving BMD and facilitating early diagnosis.

DXA scanning stands as the foundational diagnostic tool for BMD assessment, augmented by the HIV-adjusted FRAX tool for a more complete evaluation. Additionally, testing for vitamin D deficiency, likely relevant to all PLWHIV and especially the black population, is essential for maintaining skeletal health. Routine screening of both BMD and vitamin D, in pre-specified, validated regular intervals, alongside evaluations for secondary causes of bone loss, ensures early detection and effective management of potential bone health issues. Subsequently, the management approach is tailored to each individual’s unique risk profile and needs. Lifestyle adjustments, such as reducing smoking and alcohol consumption and managing comorbid conditions that contribute to bone loss, complete the preventative aspect of our plan. The integration of these lifestyle modifications, alongside dietary changes and physical activity is a critical component of the therapeutic part of our strategy, as well. The careful selection and management of ART with consideration of its impact on bone is of imminent importance, in addition to the use of pharmacological interventions to tackle progression of bone disease. The therapeutic choices should be aligned with individual patient profiles, ensuring a personalized approach to care.

Furthermore, the action plan advocates for referral to specialists—endocrinologists, rheumatologists, orthopedics, and nephrologists—under specific circumstances, such as severe osteoporosis, significant secondary causes of low BMD, or when treatment intolerance or failure occurs. Finally, a robust follow-up and patient education framework is vital for ensuring adherence and empowering patients with knowledge on bone health risks and mitigation strategies. Importantly, the intrinsic risk of bone disease posed by HIV itself should be acknowledged and integrated into patient education and management decisions. Encouraging proactive health behaviors, like regular weight-bearing exercises, healthy diet, and appropriate ART regimen adherence, is a key aspect of this patient-centric approach.

## 4. Conclusions

The subject of HIV, ART, and bone health is an actively researched and clinically significant area. As we deepen our understanding of the complex relationships between these elements, it becomes increasingly evident that addressing bone disease in people living with HIV (PWH) necessitates a nuanced and comprehensive approach. The issues discussed in this review highlight the importance of early diagnosis and well-established prevention strategies that consider both the direct impacts of the virus and its treatment on bone density, as well as the broader spectrum of risk factors unique to this population. Challenges persist, especially in ensuring access to appropriate diagnostic tools and treatments across diverse healthcare settings and in understanding the long-term implications of ART on bone health. Ongoing research is essential to unravel the mechanisms at play and to develop new strategies that can mitigate the risk of osteoporosis and fractures in this population. By adopting an integrated care approach, healthcare providers can contribute to improving the quality of life and clinical outcomes related to bone health in PWH. As the HIV-positive population ages, the urgency to address bone disease directly will only intensify, necessitating continued vigilance, innovation, and a commitment to comprehensive care.
